# Diurnal Variation and Impairment of Postprandial Glucose‐Insulin Responses and Lipid Rhythms in Simulated Shift Work

**DOI:** 10.1111/nbu.70060

**Published:** 2026-06-21

**Authors:** Ameena M. Khan Sullivan, Cheryl M. Isherwood, Hana Hassanin, Debra J. Skene, Daan R. van der Veen

**Affiliations:** ^1^ Section of Chronobiology, Faculty of Health and Medical Sciences University of Surrey Guildford UK; ^2^ Surrey Clinical Research Facility, Faculty of Health and Medical Sciences University of Surrey Guildford UK; ^3^ NIHR Royal Surrey CRF Royal Surrey NHS Foundation Trust Guildford UK

**Keywords:** blood glucose, circadian rhythm, insulin resistance, meals, postprandial period, shift work schedule

## Abstract

Shift work is associated with insulin resistance, dyslipidaemia and metabolic syndrome, but how consecutive meals at the same clock times differentially shape postprandial metabolism in day‐ and night‐shifts remains unclear. This study used a unique simulated shift work intervention with four consecutive meals and high‐resolution sampling to examine interactions of endogenous rhythms and behavioural timing on postprandial metabolism. Nine healthy non‐shift‐worker participants (age 21–33 years, 3 females) completed a strictly‐controlled laboratory protocol with a day‐shift followed by two night‐shifts of 10‐h delayed sleep and mealtimes. Participants consumed four identical, isoenergetic, mixed‐composition meals per shift. Three of four mealtimes (08:00, 18:00, 22:00) were fixed across conditions; the first and last meals were 1 h after waking and before bedtime. Plasma glucose, insulin, triglycerides, HDL‐cholesterol and total cholesterol were measured hourly, with continuous interstitial glucose monitoring. Subjective hunger and appetite were assessed throughout. Plasma lipid rhythms shifted with the 10‐h delay in sleep–wake times: acrophases delayed ~10–13 h and amplitudes lowered 46%–54% (all *p* < 0.01). Night‐shifts elevated postprandial glucose (+165 mmol/L·min, 95% CI 1.05–34.31, *p* = 0.05) and insulin (+7678.7 mmol/L·min, 95% CI 3671.02–11686.38, *p* < 0.01). Time‐of‐day effects persisted irrespective of shift, with 18:00 meals producing the highest glycaemic responses (+261 mmol/L·min, 95% CI 124.37–398.01, *p* < 0.01). Insulin dynamics did not fully compensate for elevated glycaemia in night‐shifts, indicating lower glucose tolerance and insulin sensitivity. Mistimed meals suggest a mechanism by which circadian disruption may increase metabolic disease risk.

## 1 Introduction

Approximately 8.7 million people in the UK regularly work evening or night‐time schedules (Office for National Statistics 2023). Epidemiological studies have associated chronic exposure to shift work with increased risk of metabolic syndrome (Karlsson et al. 2001), obesity (Kecklund and Axelsson 2016), type 2 diabetes (Kecklund and Axelsson 2016), cardiovascular disease (Kecklund and Axelsson 2016), depression and neurological disorders (Knutsson 2003; Wang et al. 2011). The acute effects of shift work are also burdensome and include low insulin sensitivity (Leong 2018), dyslipidaemia (Csoma and Bikov 2023), sleep deprivation (Wehrens et al. 2012), symptoms of functional bowel disorders (Knutsson and Bøggild 2010; Nojkov et al. 2010) and impaired cognition (Vlasak et al. 2022). These increased disease risks associated with shift work are partially attributed to the chronic disruption between an individual's behaviour, environment and circadian timing system.

Circadian biology functions as the body's internal timekeeping system, temporally coordinating physiology with predictable diurnal patterns in the environment. The system comprises a central pacemaker, termed the suprachiasmatic nuclei (SCN) of the hypothalamus, and peripheral molecular clocks throughout the body, including in the liver, adipose tissue and pancreas. Both the SCN and peripheral clocks can entrain to environmental signals, for example; meal schedules, the light–dark cycle and temperature fluctuations (Stokkan et al. 2001; Tuvia et al. 2021). The SCN is primarily responsive to the light–dark cycle, which in turn drives rhythmicity in pineal melatonin, which is associated with sleep onset and offset in humans. It is used as a robust biomarker of SCN circadian timing (Klerman et al. 2022).

Optimal physiological functioning occurs when circadian rhythms, driven by the central pacemaker and peripheral clocks, are aligned with diurnal rhythms driven by environmental signals (Dibner et al. 2010). Conversely, circadian desynchrony, when the relationships between environment, central pacemaker and peripheral clocks are misaligned, is detrimental to healthy physiology (Boivin et al. 2022).

Eating at inappropriate circadian times disturbs metabolic processes such as glucose metabolism and lipid clearance (Young and Bray 2007; Maury 2019). Mistimed meals, such as eating during the night when the circadian system is primed for sleep, provide conflicting signals to peripheral clocks (Gachon et al. 2017). These conflicting signals result in circadian desynchrony, which impairs the coordinated regulation of metabolic pathways. For example, the timing of insulin secretion and tissue sensitivity may become misaligned, leading to prolonged postprandial hyperglycaemia (Qian et al. 2018). Experimental models of impaired postprandial metabolism are thus important for explaining the increased risk of metabolic disease in shift workers.

Previous research has established that glucose tolerance and insulin sensitivity decline across the biological day (Wehrens et al. 2017; Leung et al. 2019). Studies have also shown that circadian misalignment akin to shift work impairs postprandial metabolism (Al‐Naimi et al. 2004; Morris et al. 2016; Qian et al. 2018; Broussard et al. 2024). Investigating the combined effects of diurnal rhythms and circadian desynchrony on postprandial metabolism has been limited by the number and timing of test meals in experimental protocols, often limited to two meals per condition (Al‐Naimi et al. 2004; Morris et al. 2016; Wehrens et al. 2017; Qian et al. 2018; Leung et al. 2019; Lundholm et al. 2024). Such schedules do not fully reflect the typical, western (Huseinovic et al. 2016) frequency and distribution of meal intake. Thus, it is necessary to investigate meal intakes typical of shift work schedules to understand the combined effects of endogenous phase and diurnal rhythm misalignment on glucose homeostasis that may not be apparent from isolated meal responses.

Our study addresses this gap by employing a simulated shift work intervention with a more typical, daily meal pattern. We served four meals per shift condition, with three meals anchored to specific clock times during both the day‐ and night‐shift conditions. We examined the extent to which biological rhythm misalignment induced by a 10‐h delay in sleep and meal timing amplifies established time‐of‐day‐dependent effects on postprandial glucose, insulin and lipids across the biological morning, afternoon, evening and night. We have used state‐of‐the‐art around‐the‐clock sampling and strict environmental control, enabling a detailed characterisation of the diurnal and postprandial variation in glucose, insulin and lipid rhythms.

## 2 Materials and Methods

### 2.1 Experimental Protocol

**FIGURE 1:**
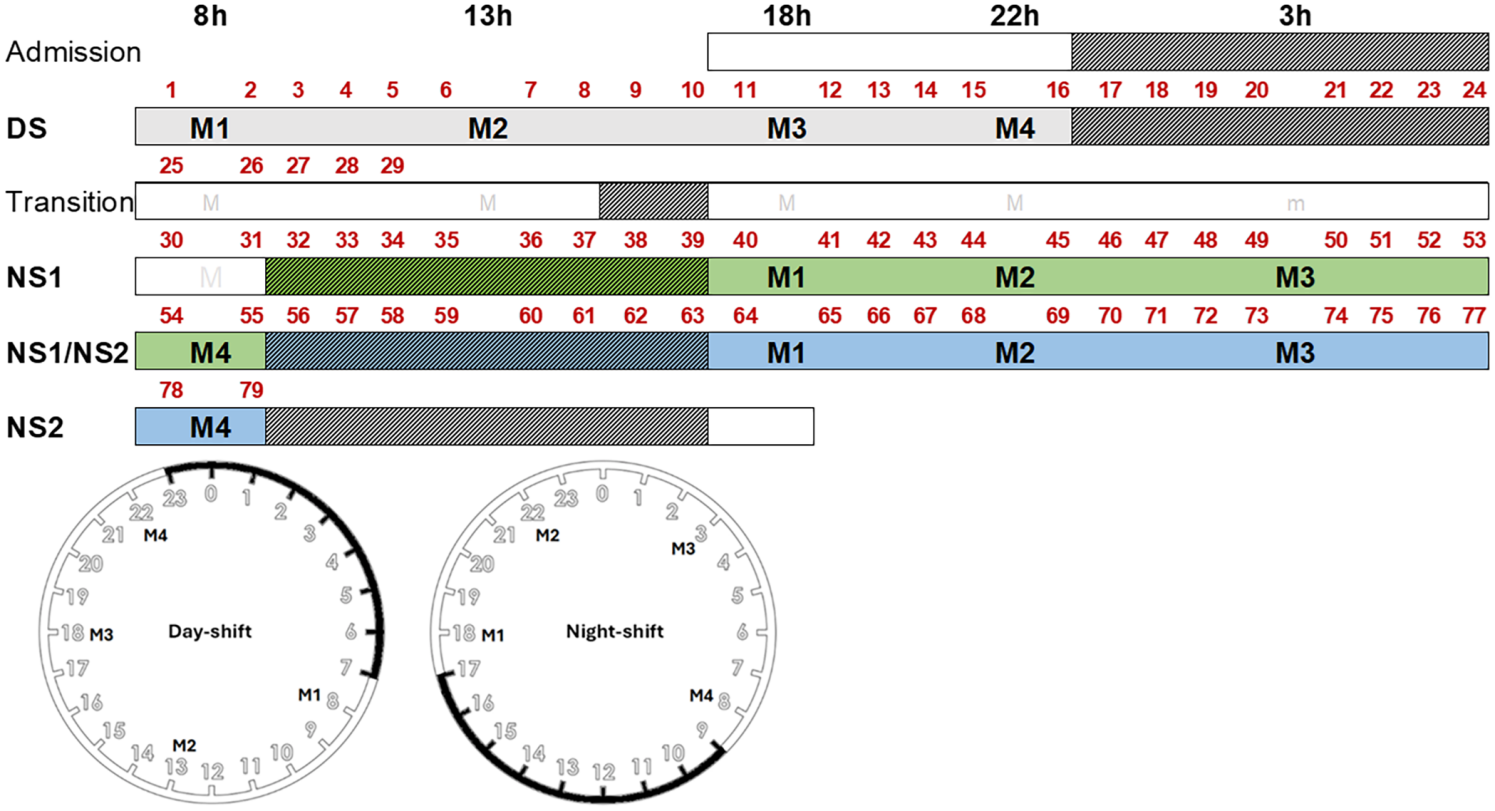
Upper: In‐laboratory study protocol showing plasma sampling, meal and sleep times. Each row represents a study day. Dark grey hatching indicates a sleep opportunity. Meals are marked M1–M4. Red numbers show serial blood sampling. Participants were admitted to the laboratory on Day 1. On the day‐shift (Day 2, grey bar), they slept from 23:00 to 07:00 and ate standardised meals at 08:00, 13:00, 18:00 and 22:00. On the transition day (Day 3, white bar), participants napped from 15:00 to 17:00 and remained awake until 09:00 on Day 4. During night‐shift 1 and night‐shift 2 (Day 4, green bar and Day 5, blue bar), they slept from 09:00 to 17:00, with sleep and meals delayed by 10 h compared to day‐shift. Lower: 24‐h clocks (hh) show 10‐h delay to mealtimes (M1–M4) and sleep opportunity (black fill) between day‐shift (Left) and night‐shift (Right).

This study received a favourable ethical opinion from the University Surrey Ethics Committee (UEC reference FHMS 21‐22 211). Informed consent, in writing, was obtained from all participants prior to the study and conformed to all standards set by the *Declaration of Helsinki version D‐1964‐01‐2013*. Participants were recruited through the Surrey Clinical Research Facility (CRF) by contacting individuals registered on the CRF database and through internal institutional and external social media advertising. Data collection occurred from August to October 2022. A 10‐day pre‐laboratory routine, a previously validated standard at the University of Surrey (Otway et al. 2011; Isherwood et al. 2017; Wehrens et al. 2017) for human chronobiology experiments, was implemented to ensure participants' stable circadian phase of entrainment and to minimise sleep deprivation prior to laboratory entry. Three days before entering the Clinical Research Facility, a continuous glucose monitor (Freestyle Libre Pro CGM, Abbott Laboratories, Illinois, USA) was attached to participants' non‐dominant arm. It sampled interstitial glucose every 15 min. During the final three‐day pre‐laboratory period, mealtimes were matched to the laboratory day‐shift (DS) mealtimes. Compliance with the pre‐laboratory routine (glucose monitors and motion/light detection devices) was checked at admission. Upon entering the laboratory, participants were habituated and undertook an 8‐h sleep opportunity. The DS started upon waking on day 2 (Figure 1, upper panel) and from this point onwards, light levels were controlled to < 5 lx during wake and 0 lx during sleep throughout the study. Following the transition day, sleep times were delayed by 10‐h in night‐shift 1 and night‐shift 2 (NS1 and NS2 respectively).

### 2.2 Meal Timing

Mealtimes were scheduled to allow comparison of postprandial responses across shift conditions by aligning the meals to both clock times and times since waking (Figure 1, lower panel). Three meals‐at 08:00, 18:00 and 22:00‐ were set to the same clock times across all shifts. Each shift included one meal at an ‘unanchored’ clock time: a meal in DS at 13:00 and a meal in the night‐shifts at 03:00 (second and third meal in DS and NS respectively). In all shifts, the first meal was served 1‐h after waking and the last meal was served 1‐h before bedtime. Detailed information about the meal protocol is included in the Supporting Information S1.

Participants ate identical meals throughout the protocol based on UK dietary guidelines (Public Health England 2016). Individual energy requirements were estimated using the Mifflin St. Jeor equation (Mifflin et al. 1990), adjusted for sex. Meals comprised 50% carbohydrates (including 15% sugars), 35% fat (11% saturated) and 15% protein. Further detail about the energy and macronutrient composition of meals is provided in Tables S1 and S2. Body weight was monitored daily to maintain stability within ±5% and water intake was unrestricted but monitored. No adjustments to meals were required during the study.

### 2.3 Blood Sampling

Hourly blood samples were collected via cannulation into lithium heparin and EDTA vacutainers from 07:30 on DS until 11:30 the following day (29 h). Collection recommenced after the transition day at 07:30 on NS1 until 08:30 on NS2 (49 h). Within 10 min of collection, samples were centrifuged at 1620 × *g* at 4°C for 10 min. Samples were then frozen at −80°C within 30 min of collection. Plasma glucose, triglycerides, total cholesterol (TC) and high‐density‐lipoprotein cholesterol (HDL‐C) concentrations were measured using the PENTRA c400 Clinical Chemistry Analyser (HORIBA ABX SAS, Montpellier, France). Insulin was quantified using ELISA kits (EZHI‐14 K, Merck KGgA, Darmstadt, Germany). Plasma melatonin was quantified using Liquid Chromatography–Tandem Mass Spectrometry (LC–MS/MS) procedures (University Medical Centre Groningen, Netherlands (Van Faassen et al. 2017)). Subjective hunger, fullness, desire to eat and cravings for savoury, salty, sweet and fatty foods were assessed using visual analogue scales (VAS) 2‐ and 1‐h before and 1 and 2 hrs after each meal. Continuous interstitial glucose monitoring continued during the laboratory study as during the pre‐laboratory period.

### 2.4 Participants

Study participants (6 males and 3 females) were healthy, with mean (±SD) age 28.8 (±3.1 years) and BMI 25 (±2 kg/m^2^). Inclusion criteria included the requirement of regular sleep schedules and normal daytime sleepiness (Epworth Sleepiness Scale ≤ 10), diurnal preference (Horne‐Östberg score 30–70) and sleep quality (Pittsburgh Sleep Quality Index ≤ 5) and females taking a combined oral contraceptive. Exclusion criteria included shift work or travel across time zones within the last 3 months, use of supplements or medications affecting sleep and circadian biology and no medical conditions or taking medications known to affect study outcomes.

### 2.5 Outcome Measures

The hourly concentration profiles of plasma melatonin, insulin, glucose, triglycerides, HDL‐C and TC were determined. Non‐HDL‐C concentrations were calculated by subtracting HDL‐C from TC concentrations (mM) for each timepoint. Incremental Area Under the Curve (iAUC) using the trapezoidal rule was calculated for plasma glucose, insulin, triglycerides and interstitial glucose postprandial responses (Flanagan and Fielding 2022). Pre‐meal concentrations were measured 30 minutes before a meal. iAUCs had a 180‐min duration, from +30 to +210 minutes after the meal. Questions for VAS appetite ratings (Table 5) were presented on a hardcopy 100 mm scale, anchored with ‘Not at all’ on the left and ‘Extremely’ on the right. These were measured and converted to centimetres (cm).

### 2.6 Statistical Analysis

All data were plotted by clock time in GraphPad Prism (v10.0.1218), for visual inspection. Distributions were tested for normality using the Shapiro–Wilk test. Dim Light Melatonin Onset (DLMO), defined as the time at which melatonin concentrations first rise above a threshold value under dim‐light conditions, was calculated using validated 25% threshold methods, previously described (Sletten et al. 2009; Wehrens et al. 2017; Bello et al. 2024). Differences in DLMO were tested with a mixed‐effects‐ANOVA in GraphPad Prism (v10.0.1218). Cosine curves were fitted to 24‐h concentration data using our established linear mixed‐effects cosine model (cosinor analysis) in MatLab (R2023a Update 5) (Bello et al. 2024). Rhythm amplitudes, representing the magnitude of rhythmic variation around the fitted mean and acrophases, defined as the timing of the peak of the fitted 24‐h rhythm, were derived from cosinor analysis. A 24‐h timeseries from 08:30 to 07:30 h, per shift, was used for analysis. Differences in acrophases and amplitudes were tested with unpaired two‐tailed *t*‐tests in GraphPad Prism (v10.4.2633).

The effect of shift condition on average concentrations of plasma glucose, insulin, triglycerides, TC, HDL‐C and non‐HDL‐C was tested with a linear mixed‐effects model using the lme4 package (v1.1‐36) in R (v4.4.2), using the same 24‐h data as in the cosinor analysis. Fixed effects were shift (categorical), time of day (continuous) and their interaction.

Linear mixed‐effects models and subsequent pairwise comparisons for plasma glucose, insulin, triglycerides and interstitial glucose iAUC were fitted using the lme4 package (v1.1‐36) and emmeans package (v.1.11.2) in R (v4.4.2). Fixed effects included time of meal and shift (both categorical) and their interaction. Pairwise comparisons between estimated marginal means were conducted across all combinations of shift and time of day. *p*‐values were adjusted for multiple testing using Tukey HSD test and all comparisons were based on model‐derived estimates. VAS ratings were analysed using linear mixed‐effects models with fixed effects of shift (categorical) and time since wake (continuous).

In all models, participants were included as a random intercept. All model assumptions were evaluated using residual diagnostics. All data are presented as group mean ± standard error of the mean (SEM), unless standard deviation (SD) is indicated. ‘Time’ refers to local clock time of day (HH:mm), reported in British Summer Time (UTC + 01:00).

## 3 Results

### 3.1 Dim Light Melatonin Onset (DLMO) Does Not Change During Night‐Shifts

To test whether melatonin phase was shifted in response to the 10‐h delay in meal and sleep times, we examined changes in DLMO between shifts. Mean DLMO occurred at 21:41 ± 32 min (±SD) in DS, 22:17 ± 40 min in NS1 and 22:05 ± 60 min in NS2. It did not differ significantly between shifts (*p* = 0.14) or participants (*p* = 0.11).

### 3.2 24‐h Mean Metabolic Biomarker Concentrations Are Lower in Night‐Shifts

We determined the mean 24‐h concentrations of glucose, insulin, TC, HDL‐C, non‐HDL‐C and triglycerides during DS, NS1 and NS2. Plasma, TC, HDL‐C and non‐HDL‐C concentrations were significantly lower in night‐shifts compared to DS (NS1: TC, HDL‐C and non‐HDL‐C all *p*‐values < 0.01; NS2, all *p* < 0.01). [Correction added on August 4, 2026 after first online publication: The word “glucose” is removed from the previous sentence in this version.] Triglyceride concentrations were significantly lower in NS2 compared with DS (*p* = 0.01), but not in NS1 (*p* = 0.09). Average 24‐h plasma insulin concentrations did not differ significantly between shifts. Mean 24‐h concentrations, corresponding *p‐*values and confidence intervals are provided in Table 1.

**TABLE 1:** Model‐derived mean concentrations of analytes by shift condition, adjusted for random effects. Differences in average concentrations of analytes in night‐shifts compared to day‐shift are estimated by a linear mixed‐effects model. Data are presented as mean concentration [95% confidence interval].

	DS mean [95% CI]	NS1 mean [95% CI]	NS2 mean [95% CI]	DS vs. NS1 [95% CI]	*p*‐value	DS vs. NS2 [95% CI]	*p*‐value
Glucose	5.99 [5.75–6.23]	6.28 [6.04–6.52]	6.41 [6.16–6.67]	0.29 [0.05–0.54]	**0.02**	0.42 [0.16–0.68]	**< 0.01**
Insulin	38.35 [26.25–50.45]	39.02 [26.90–51.15]	34.81 [22.46–47.16]	0.68 [−7.15–8.50]	0.87	−3.54 [−11.82–4.73]	0.40
Triglycerides	1.41 [1.04–1.78]	1.36 [0.99–1.73]	1.32 [0.95–1.69]	−0.06 [−0.13–0.01]	0.09	−0.09 [−0.17 – −0.02]	**0.01**
TC	4.65 [4.20–5.09]	4.33 [3.89–4.78]	4.30 [3.86–4.75]	−0.31 [−0.38 – −0.25]	**< 0.01**	−0.34 [−0.41 – −0.27]	**< 0.01**
HDL‐C	1.09 [0.87–1.30]	1.03 [0.81–1.24]	1.03 [0.82–1.25]	−0.06 [−0.08 – −0.04]	**< 0.01**	−0.05 [−0.08 – −0.03]	**< 0.01**
Non‐HDL‐C	3.56 [3.11–4.01]	3.30 [2.86–3.75]	3.26 [2.81–3.71]	−0.25 [−0.30 – −0.20]	**< 0.01**	−0.3 [−0.35 – −0.25]	**< 0.01**

*Note:* Insulin is presented as μU/mL, all other units are mmol/L. Bold signifies significant *p*‐value (< 0.05).

Abbreviations: 95% CI, 95% confidence intervals; DS, day‐shift; HDL‐C, high‐density‐lipoprotein cholesterol; non‐HDL‐C, non‐high‐density‐lipoprotein cholesterol; NS1, night‐shift 1; NS2, night‐shift 2; TC, total cholesterol.

### 3.3 24‐h Rhythms of Plasma Glucose, Insulin and Lipids Phase Delay During Night‐Shifts

**FIGURE 2:**
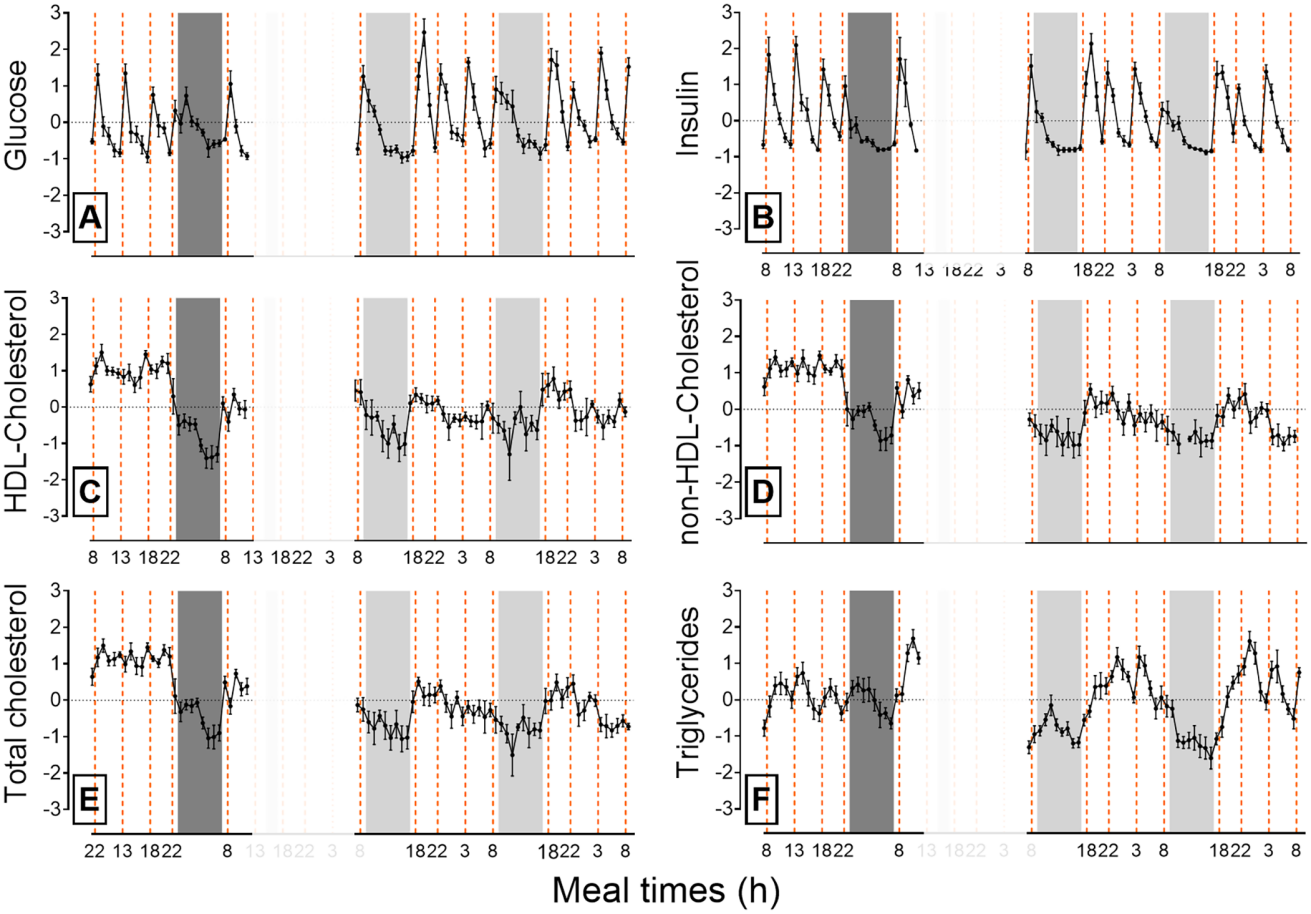
Concentration time series of plasma glucose [A], insulin [B], high‐density‐lipoprotein cholesterol (HDL‐C) [C], non‐high‐density‐lipoprotein‐cholesterol (non‐HDL‐C) [D], total cholesterol [E] and triglycerides [F]. Data are presented as z‐scored group mean ± SEM. *X*‐axis indicates clock time of meals, marked by orange vertical dashed lines. Dark grey band shows day‐shift sleep opportunity; light grey band shows night‐shift sleep opportunities.

Next, we examined whether the delay in sleep and meal timings altered the 24‐h temporal dynamics of plasma glucose, insulin and lipid concentrations (Figure 2) using linear mixed‐effects cosine models. Insulin, TC, HDL‐C and non‐HDL‐C were rhythmic in all shifts (Table 2). Triglycerides were rhythmic during NS1 (*p* < 0.01) and NS2 (*p* < 0.01), while plasma glucose exhibited rhythmicity in NS1 only (*p* < 0.01).

**TABLE 2:** Peak time (acrophase) and amplitudes of 24‐h rhythms of plasma glucose, insulin and lipids.

	Cosine *p*‐value	Acrophase [95% CI]	Amplitude [95% CI]
DS			
Glucose	n.s.		
Insulin	**< 0.01**	15:00 [13:18–16:42]	17.83 [8.96–26.70]
TC	**< 0.01**	15:12 [14:24–16:00]	0.34 [0.21–0.46]
HDL‐C	**< 0.01**	15:00 [14:06–15:54]	0.08 [0.06–0.11]
Non‐HDL‐C	**< 0.01**	15:18 [14:24–16:12]	0.26 [0.15–0.36]
Triglycerides	n.s.		
NS1			
Glucose	**< 0.01**	03:42 [02:24–05:06]	0.93 [0.56–1.30]
Insulin	**< 0.01**	04:06 [03:00–05:12]	29.50 [18.34–40.70]
TC	**< 0.01**	01:30 [23:36–03:30]	0.16 [0.02–0.29]
HDL‐C	**< 0.01**	03:42 [01:12–06:18]	0.04 [0.01–0.07]
Non‐HDL‐C	**< 0.01**	01:00 [22:54–03:06]	0.12 [0.03–0.22]
Triglycerides	**< 0.01**	01:48 [00:48–02:48]	0.34 [0.23–0.44]
NS2			
Glucose	n.s.		
Insulin	**< 0.01**	03:36 [02:00–04:42]	24.00 [8.72–39.32]
TC	**< 0.01**	21:36 [19:18–23:54]	0.17 [0.06–0.28]
HDL‐C	**< 0.01**	20:06 [16:54–23:24]	0.04 [0.01–0.06]
Non‐HDL‐C	**< 0.01**	21:48 [19:36–00:00]	0.14 [0.05–0.22]
Triglycerides	**< 0.01**	02:18 [01:30–03:12]	0.43 [0.31–0.56]

*Note:* Cosine curves fitted to data with linear mixed‐effects model. Acrophase is presented in hh:mm. Units of amplitude are mM for glucose, TC, HDL‐C, non‐HDL‐C and triglycerides. Insulin amplitude is measured in μU/mL. Bold signifies significant *p‐*value (< 0.05).

Abbreviations: HDL‐C, high‐density‐lipoprotein cholesterol; n.s., not significant; non‐HDL‐C, non‐high‐density‐lipoprotein cholesterol; TC, total cholesterol.

In DS, insulin amplitude was 17.8 μU/mL, increasing by 66% to 29.5 μU/mL in NS1 (*p* < 0.01). The change in NS2 (24.0 μU/mL) was not statistically significant (*p* = 0.06). By contrast, TC, HDL‐C and non‐HDL‐C amplitudes were significantly lower in the night‐shifts compared to the day‐shift (all *p*‐values < 0.01, Table 3). Specifically, TC amplitude was 54% lower in NS1 (0.16 mM) and 50% lower in NS2 (0.17 mM) than in DS (0.34 mM). HDL‐C amplitude was 50% lower in NS1 (0.04 mM) and 50% lower in NS2 (0.04 mM) than in DS (0.08 mM). Non‐HDL‐C amplitude was 52% lower in NS1 (0.12 mM) and 47% lower in NS2 (0.14 mM) than in DS (0.26 mM). Triglycerides were not rhythmic in DS but showed amplitudes of 0.34 mM and 0.43 mM in NS1 and NS2, respectively.

**TABLE 3:** Differences in acrophase and rhythm amplitude of rhythmic analytes in night‐shifts compared to day‐shift. Data are presented as difference between group mean acrophase (±SEM) in either night‐shift 1 or night‐shift 2 compared to day‐shift.

	DS vs. NS1				DS vs. NS2			
	Difference between mean acrophase ± SEM	*p*‐value	Difference between mean amplitude ± SEM	*p*‐value	Difference between mean acrophase ± SEM	*p*‐value	Difference between mean amplitude ± SEM	*p*‐value
Insulin	13.1 ± 0.35	**< 0.01**	11.69 ± 2.40	**< 0.01**	12.4 ± 0.37	**< 0.01**	6.19 ± 3.01	0.06
TC	10.3 ± 0.36	**< 0.01**	−0.18 ± 0.03	**< 0.01**	6.4 ± 0.42	**< 0.01**	−0.17 ± 0.03	**< 0.01**
HDL‐C	12.7 ± 0.46	**< 0.01**	−0.04 ± 0.01	**< 0.01**	5.1 ± 0.58	**< 0.01**	−0.04 ± 0.01	**< 0.01**
Non‐HDL‐C	9.7 ± 0.39	**< 0.01**	−0.14 ± 0.02	**< 0.01**	6.5 ± 0.41	**< 0.01**	−0.12 ± 0.02	**< 0.01**

*Note:* Differences tested by two‐tailed, unpaired *t*‐tests. Mean difference in acrophase is presented as decimal time (h.m). Mean difference in amplitude is presented as mM for TC, HDL‐C, non‐HDL‐C and μU/mL for insulin. Bold signifies significant *p*‐value (< 0.05).

Abbreviations: df, degrees of freedom; DS, day‐shift; HDL_C, high‐density‐lipoprotein cholesterol; non‐HDL‐C, non‐high‐density‐lipoprotein cholesterol; NS1, night‐shift 1; NS2, night‐shift 2; SEM, standard error of mean; TC, total cholesterol.

**FIGURE 3:**
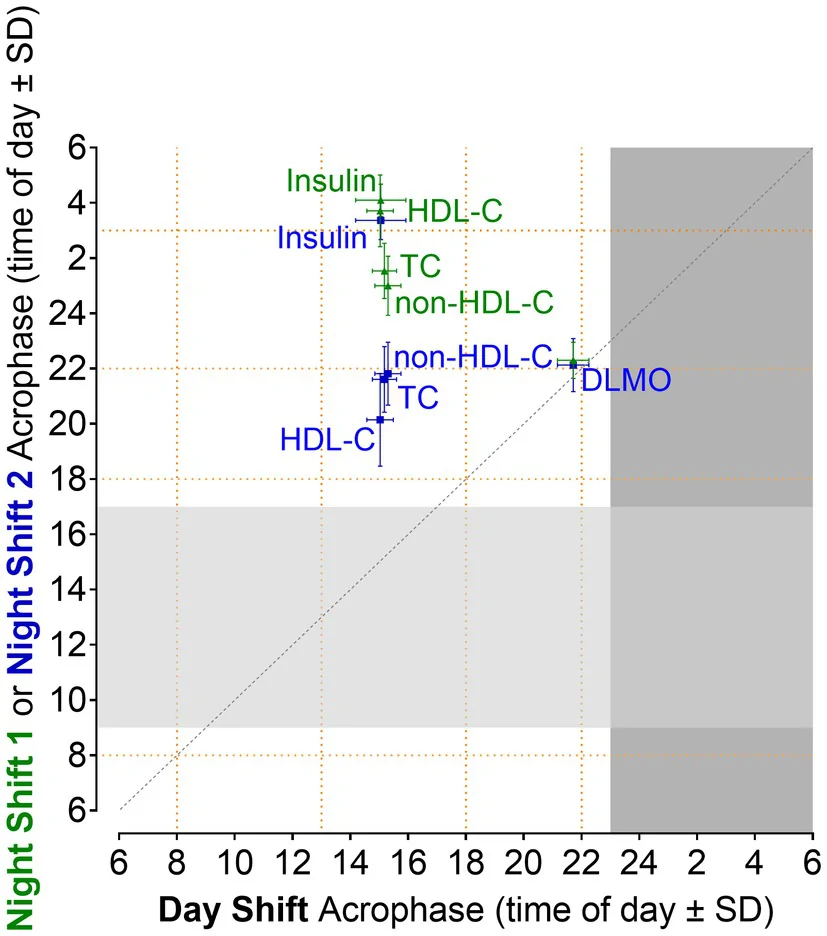
Acrophase times of analytes in day‐shift (*X*‐axis) and NS1 (*Y*‐axis, green symbol) and NS2 (*Y*‐axis, blue symbol). Timing of the peak of the 24‐h rhythm (acrophase) for plasma insulin and lipids in day‐shift and night‐shift conditions. Each symbol represents mean acrophase ± SEM. The dashed diagonal line indicates where acrophases would fall if their timing remained unchanged between day and night‐shifts. Green symbols compare DS to NS1, blue symbols compare DS to NS2. Orange bars mark mealtimes and grey shading indicates sleep opportunity. Dim Light Melatonin Onset (DLMO) is included as a SCN phase reference. Time (hh) is in 24 h (24 = midnight). Markers deviating from the dashed diagonal line reflect acrophase differences between day and night‐shift conditions.

Insulin, TC, HDL‐C and non‐HDL‐C rhythm mean acrophases occurred in the afternoon in DS, between 15:00–15:18 (Table 2). During the night‐shifts, the acrophases were significantly delayed compared to DS, their mean acrophases occurring between 01:00–04:06 in NS1 and 20:06–03:36 in NS2 (Figure 3). In NS1, insulin phase delayed by 13 h 6 m, TC by 10 h 18 m, HDL‐C by 12 h 42 m and non‐HDL‐C by 9 h 42 m (all *p* < 0.01). In NS2, insulin phase delayed by 12 h 36 m, TC by 6 h 24 m, HDL‐C by 5 h 6 m and non‐HDL‐C by 6 h 30 m compared to DS (all *p* < 0.01).

### 3.4 Postprandial Glucose and Insulin Dynamics Are Prolonged in Night‐Shifts

**FIGURE 4:**
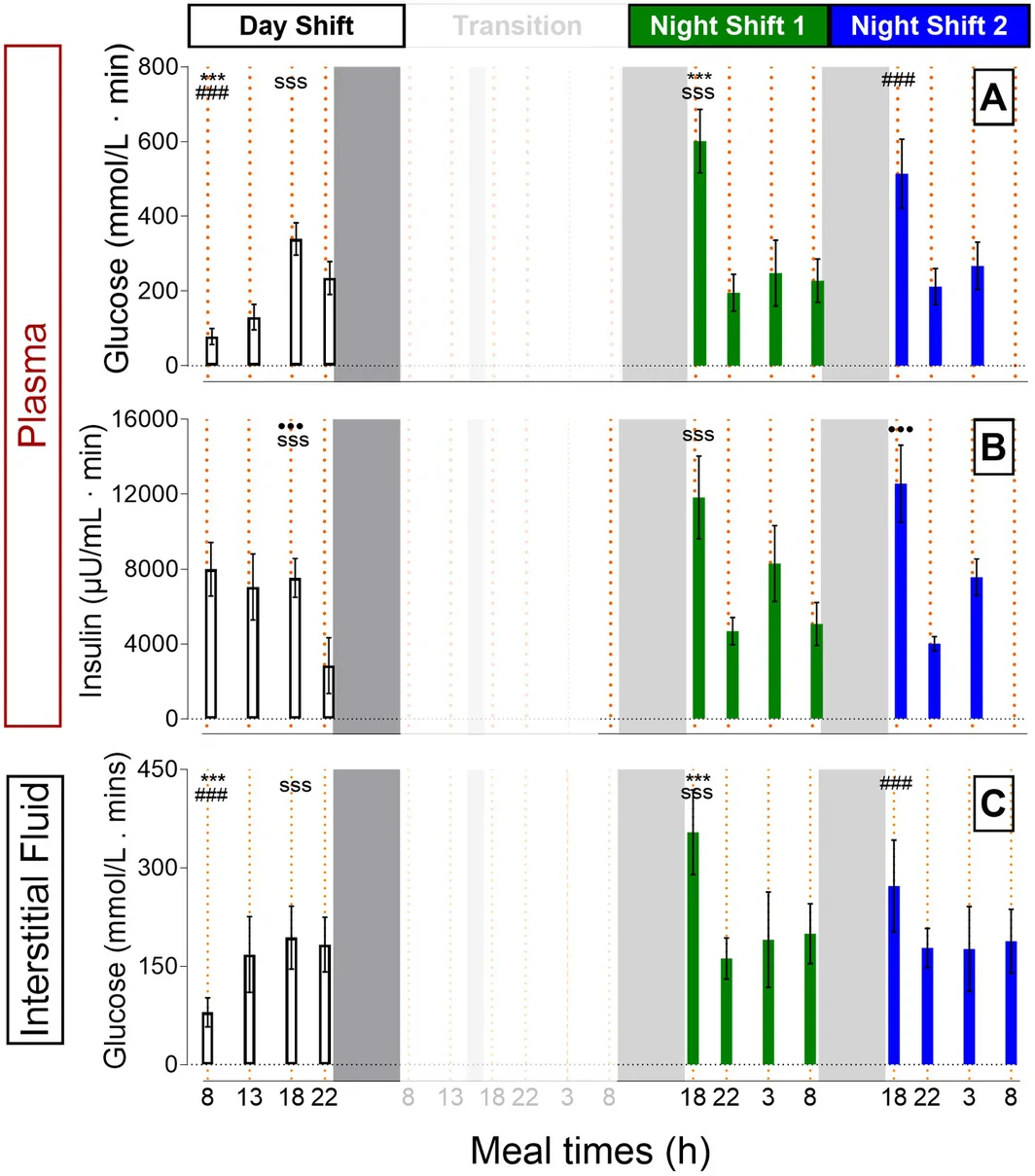
Plasma Glucose and Insulin, and Interstitial Glucose Responses (iAUC ± SEM) to meals during Day‐ and Night‐Shift Conditions. *Y*‐axis shows (A) plasma glucose, (B) plasma insulin and (C) interstitial glucose responses. *X*‐axis indicates clock time of meals, marked by orange vertical dashed lines. Dark grey band shows day‐shift sleep opportunity; light grey band shows night‐shift sleep opportunities. ***Compares DS and NS1 first meal since wake. ^###^Compares DS and NS2 first meal since wake. ^sss^Compares DS and NS1 meal at 18:00. ^●●●^Compares DS and NS2 meal at 18:00. All *p* < 0.05.

To assess whether an effect of shift on postprandial responses varied by time of day and time since waking, we determined the incremental area under the curve (iAUC) for plasma glucose, insulin and triglycerides and interstitial glucose, after each meal; as shown in Figure 4. We analysed the iAUC for meals consumed at fixed clock times (08:00, 18:00 and 22:00), corresponding to either +1 or + 15 h since waking in day‐ and night‐shifts. Below we report the model‐estimated differences in iAUCs between shifts and mealtimes.

### 3.5 Plasma Glucose iAUC Varied Between Shifts and Mealtimes

Glucose responses in NS1 were greater than in DS (164.8 mmol/L·min, 95% CI: 1.1 to 328.5, *p* = 0.049). Responses to 18:00 and 22:00 were greater compared to 08:00 (18:00: 261.2 mmol/L·min, 95% CI: 124.4 to 398.0, *p* < 0.01; 22:00: 163.0 mmol/L·min, 95% CI: 21.6 to 304.3, *p* = 0.03). Full results are shown in Table 4.

**TABLE 4:** Estimates of main effects of mealtimes (18, 22 h vs. 8 h) and shift (DS vs. NS1, DS vs. NS2) and interactions effects of mealtimes × shifts on incremental area under the curve (iAUC) [95% confidence interval].

Fixed effects	Plasma glucose iAUC [95% CI]	*p*‐value	Plasma insulin iAUC [95% CI]	*p*‐value	Interstitial glucose iAUC [95% CI]	*p*‐value	Plasma triglyceride iAUC [95% CI]	*p*‐value
(Intercept)	78.39 [−25.04–181.82]	0.13	7999.51 [5131.32–10867.69]	**< 0.01**	60 [−22.78–142.78]	0.15	62.43 [34.95–89.91]	**< 0.01**
Shift [NS1]	164.77 [1.05–328.49]	**0.05**	−3206.83 [−6271.76 – −141.90]	**0.04**	109.67 [24.12–195.22]	**0.01**	−137.61 [−183.58 – −91.63]	**< 0.01**
Shift [NS2]	−27.73 [−179.26–123.81]	0.72	−3.75 [−3490.85–3483.35]	0.99	95.33 [9.78–180.88]	**0.03**	33.38 [−10.68–77.44]	0.13
ToD [18 h]	261.19 [124.37–398.01]	**< 0.01**	−468.11 [−2993.55–2057.34]	0.71	89.83 [4.28–175.38]	**0.04**	−39.07 [−77.92 – −0.21]	**0.05**
ToD [22 h]	162.96 [21.60–304.31]	**0.03**	−4182.31 [−6909.65 – −1454.97]	**< 0.01**	82.75 [−2.80–168.30]	0.06	−34.96 [−76.50–6.58]	0.10
Shift [NS1] × ToD [18 h]	94.5 [−125.36–314.35]	0.39	7678.7 [3671.02–11686.38]	**< 0.01**	63.92 [−57.07–184.90]	0.30	164.09 [102.13–226.05]	**< 0.01**
Shift [NS2] × ToD [18 h]	203.84 [−6.04–413.72]	0.06	4540.81 [172.50–8909.12]	**0.04**	0.75 [−120.24–121.74]	0.99	27.88 [−32.67–88.43]	0.36
Shift [NS1] × ToD [22 h]	−207.37 [−426.55–11.81]	0.06	3584.88 [−617.98–7787.75]	0.09	−117.83 [−238.82–3.15]	0.06	143.39 [79.71–207.06]	**< 0.01**
Shift [NS2] × ToD [22 h]					−77.17	0.21		

*Note:* Bold signifies significant *p*‐value (< 0.05). iAUC for plasma glucose, interstitial glucose and plasma triglycerides presented in mmol/L•min; plasma insulin is presented as μU/mL•min.

Abbreviations: DS, day‐shift; NS1, night‐shift 1; NS2, night‐shift 2; ToD, time of day’.

In terms of time‐of‐day variation, in DS, the 18:00 meal elicited the largest response‐ which was significantly larger compared to 08:00 (261.2 mmol/L·min, 95% CI: 44.5 to 477.8, *p* < 0.01). There was no significant difference between the 18:00 and 22:00 meal responses, nor the 08:00 and 22:00 responses (all *p* > 0.05).

We similarly observed the maximal postprandial response to 18:00 during night‐shifts. The response to the 18:00 meal in NS1 was significantly larger compared to 08:00 (355.7 mmol/L·min, 95% CI: 81.7 to 629.7, *p* < 0.01). In both NS1 and NS2, the responses to 18:00 were larger compared to 22:00 (NS1: 400.1 mmol/L·min, 95% CI: 161.4 to 638.8, p = < 0.01; NS2: 302.1 mmol/L·min, 95% CI: 56.4 to 547.7, *p* < 0.01).

The 18:00 response in NS1 was significantly elevated compared to the same timepoint in DS (difference: 259.3 mmol/L·min, 95% CI: 26.0 to 492.5, *p* = 0.02), as shown in Figure 4. When comparing the first meals since wake (DS: 08:00; NS1/NS2: 18:00), postprandial responses were significantly greater in the night‐shifts than in the day‐shift (NS1 vs. DS: 520.5 mmol/L·min, 95% CI: 287.2 to 753.7, *p* < 0.01; NS2 vs. DS: 437.3 mmol/L·min, 95% CI: 203.9 to 670.7, *p* < 0.01).

### 3.6 Plasma Insulin iAUC Was Different Between Shifts and Mealtimes

Overall insulin responses were significantly lower in NS1 than in the DS (−3206.8 μU/mL·min, 95% CI: −6271.8 to −141.9, *p* = 0.04). In all shifts, responses to 22:00 were significantly lower compared to 08:00 (−4182.3 μU/mL·min, 95% CI: −6909.7 to −1455.0, *p* < 0.01). Responses to meals at 18:00 in NS1 and NS2 were greater compared to the first meal in DS (NS1: 7678.7 μU/mL·min, 95% CI: 3671.0 to 11686.4, *p* < 0.01 and NS2: 4540.8 μU/mL·min, 95% CI: 172.5 to 8909.1, *p* = 0.04).

In NS1, the 18:00 meal insulin response was significantly greater than both 08:00 (difference: 7210.6 μU/mL·min, 95% CI: 2264.8 to 12156.4, *p* < 0.01) and 22:00 (7808.0 μU/mL·min, 95% CI: 3371.4 to 12244.7, *p* < 0.01). A similar pattern was observed in NS2, where the 18:00 meal elicited a significantly greater response compared to 22:00 (8255.0 μU/mL·min, 95% CI: 2814.7 to 13695.3, *p* < 0.01).

As shown in Figure 4, the insulin response at 18:00 in the night‐shifts was significantly elevated compared to the day‐shift (NS1 vs. DS: 4471.9 μU/mL·min, 95% CI: 302.1 to 8641.6, *p* = 0.03; NS2 vs. DS: 4537.1 μU/mL·min, 95% CI: 179.9 to 8894.2, *p* = 0.04).

### 3.7 Interstitial Glucose iAUC Differed Across Shifts and Mealtimes

Interstitial glucose iAUC responses were significantly greater in NS1 compared to DS (109.7 mmol/L·min, 95% CI: 24.1 to 195.2, *p* = 0.01) and in NS2 relative to DS (95.3 mmol/L·min, 95% CI: 9.8 to 180.9, *p* = 0.03). Responses to 18:00 were significantly higher than 08:00 (89.8 mmol/L·min, 95% CI: 4.3 to 175.4, *p* = 0.04). Full model results are presented in Table 4.

In NS1, the 18:00 meal produced a significantly larger glucose response than both 08:00 (difference 153.8 mmol/L·min, 95% CI: 16.0 to 291.5, *p* = 0.02) and 22:00 meals (188.8 mmol/L·min, 95% CI: 51.1 to 326.5, *p* < 0.01). The 18:00 response in NS1 was significantly greater than at the same clock time in the day‐shift (173.6 mmol/L·min, 95% CI: 35.9 to 311.3, *p* < 0.01). When responses were compared according to their meal order, the 18:00 meal in NS1 was larger than the first meal (08:00) in DS (first meal since waking: 263.4 mmol/L·min, 95% CI: 125.7 to 401.1, *p* < 0.01) (Figure 4). A comparable pattern was observed in NS2, where the first meal since waking (18:00) elicited a significantly greater response than the corresponding first meal (08:00) in the day‐shift (185.9 mmol/L·min, 95% CI: 48.2 to 323.6, *p* < 0.01).

### 3.8 Plasma Triglycerides

**FIGURE 5:**
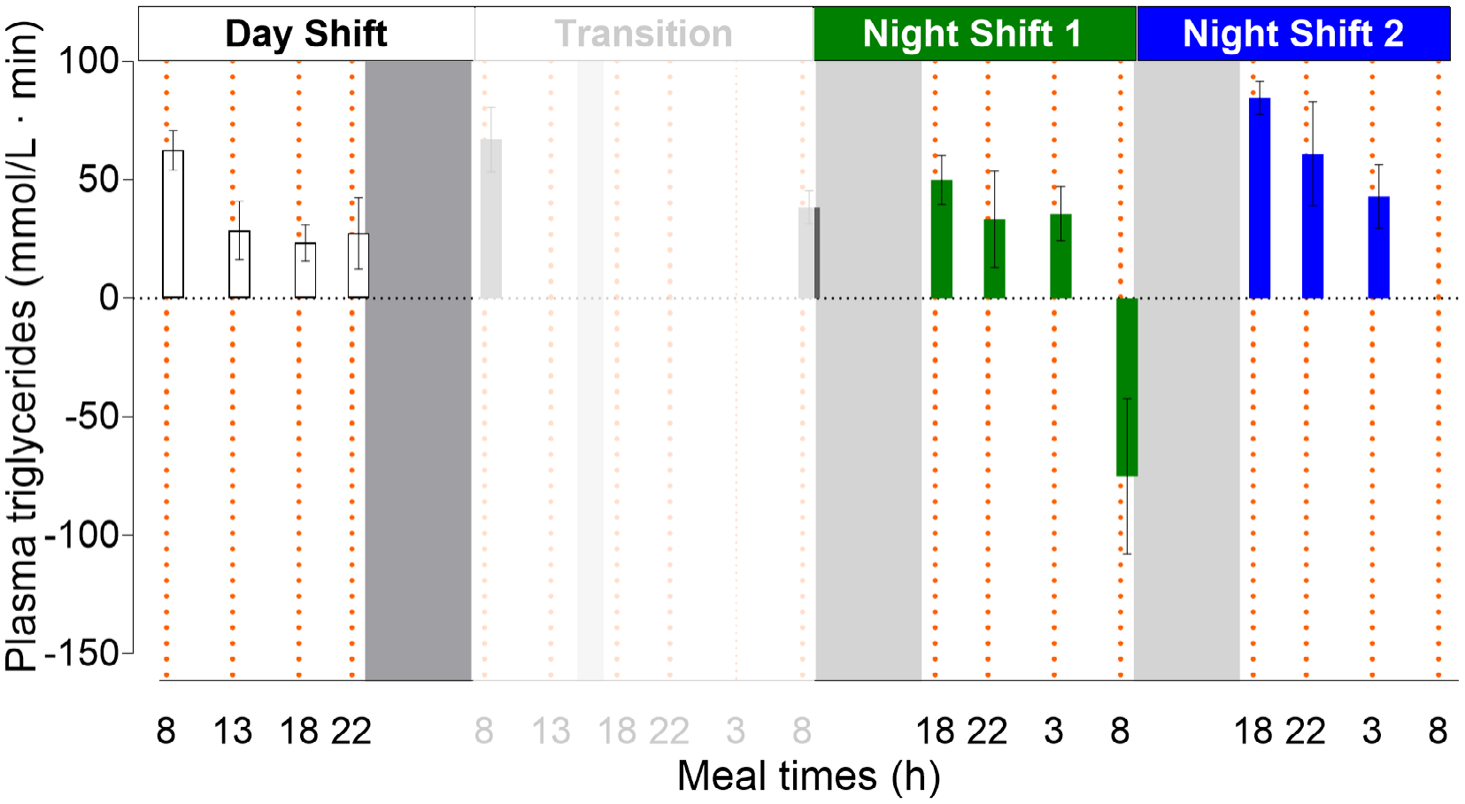
Incremental area under the curve (iAUC) for plasma triglycerides. *X*‐axis indicates clock time of meals (h), marked by orange vertical dashed lines. Dark grey band shows day‐shift sleep opportunity; light grey band shows night‐shift sleep opportunities.

In NS1, the 08:00 meal elicited a significantly lower plasma triglyceride response (iAUC) compared to all other time points across shifts (all *p* < 0.01), falling below pre‐meal triglyceride concentrations during the 180‐min postprandial period. There were no other significant pairwise differences within and between shifts (Figure 5). NS1 08:00 iAUC was significantly lower than DS 08:00 (−137.6 mmol/L·min, 95% CI: −211.0 to −64.2, *p* < 0.01), DS 18:00 (−98.5 mmol/L·min, 95% CI: −171.9 to −25.2, *p* < 0.01), DS 22:00 (−102.6 mmol/L·min, 95% CI: −179.7 to −25.6, *p* < 0.01), NS1 18:00 (−125.0 mmol/L·min, 95% CI: −202.2 to −47.8, *p* < 0.01), NS1 22:00 (−108.4 mmol/L·min, 95% CI: −185.3 to −31.6, *p* < 0.01), NS2 18:00 (−159.8 mmol/L·min, 95% CI: −236.7 to −82.9, *p* < 0.01) and NS2 22:00 (−136.0 mmol/L·min, 95% CI: −212.9 to −59.2, *p* < 0.01).

### 3.9 Subjective Ratings of Hunger and Appetite

Subjective ratings of hunger, desire to eat, fullness and appetite were analysed using linear mixed‐effects models, model estimates are detailed in Table 5. Significant interactions between time since wake and shift condition were observed for hunger (NS1: *p* < 0.01; NS2: *p* = 0.01) and desire to eat (NS1: *p* < 0.01; NS2: *p* < 0.01). Hunger and desire to eat declined over time since wake in both night‐shift conditions relative to DS. For hunger, the decline was −0.16 cm (95% CI: −0.26 to −0.06) in NS1 and −0.13 cm (95% CI: −0.23 to −0.03) in NS2. For desire to eat, the decline was −0.15 cm (95% CI: −0.2 to −0.07) in NS1 and −0.12 cm (95% CI: −0.21 to −0.04) in NS2. Fullness increased over time in NS2 compared to DS (0.13 cm, 95% CI: 0.03 to 0.24, *p* = 0.01).

**TABLE 5:** Estimated differences in ratings of subjective hunger (cm) and appetite from linear mixed‐effects model showing the main effects of time since wake and shift condition (DS vs. NS1, DS vs. NS2) and interactions between time since wake and shift conditions (NS1, NS2).

	Intercept estimate [95% CI]	*p*‐value	Time since wake estimate [95% CI]	*p*‐value	NS1 estimate [95% CI]	*p*‐value	NS2 estimate [95% CI]	*p*‐value	TsW × NS1 estimate [95% CI]	*p*‐value	TsW × NS2 estimate [95% CI]	*p*‐value
Hunger	**3.54 [2.18–4.89]**	**< 0.01**	0.07 [−0.01–0.14]	0.07	**1.06 [0.05–2.08]**	**0.04**	0.64 [−0.38–1.65]	0.22	**−0.16 [−0.26 – −0.06]**	**< 0.01**	**−0.13 [−0.23 – −0.03]**	**0.01**
Fullness	**5.04 [3.95–6.12]**	**< 0.01**	−0.05 [−0.12–0.03]	0.23	−0.44 [−1.51–0.63]	0.42	−1.03 [−2.10–0.04]	0.06	0.07 [−0.03–0.18]	0.18	**0.13 [0.03–0.24]**	**0.01**
Prospective consumption	**4.54 [3.14–5.95]**	**< 0.01**	0.03 [−0.03–0.09]	0.38	0.77 [−0.11–1.65]	0.09	0.16 [−0.72–1.05]	0.72	**−0.15 [−0.24 – −0.07]**	**< 0.01**	**−0.12 [−0.21 – −0.04]**	**0.01**
Thirst	**5.96 [4.57–7.34]**	**< 0.01**	**−0.16 [−0.22 – −0.11]**	**< 0.01**	**−1.41 [−2.19 – −0.62]**	**< 0.01**	**−1.04 [−1.82 – −0.26]**	**0.01**	**0.12 [0.04–0.20]**	**< 0.01**	0.05 [−0.02–0.13]	0.18
Savoury	**5.48 [3.83–7.13]**	**< 0.01**	0.06 [−0.01–0.12]	0.08	**0.94 [0.05–1.82]**	**0.04**	0.41 [−0.48–1.31]	0.37	**−0.15 [−0.24 – −0.07]**	**< 0.01**	**−0.13 [−0.22 – −0.05]**	**< 0.01**
Salty	**5.09 [3.40–6.79]**	**< 0.01**	0.05 [−0.01–0.11]	0.13	0.58 [−0.31–1.47]	0.20	0.21 [−0.69–1.10]	0.65	**−0.15 [−0.23 – −0.06]**	**< 0.01**	**−0.12 [−0.21 – −0.03]**	**0.01**
Sweet	**4.77 [3.03–6.50]**	**< 0.01**	0.06 [−0.00–0.12]	0.07	0.73 [−0.14–1.60]	0.10	0.23 [−0.63–1.09]	0.60	**−0.15 [−0.24 – −0.06]**	**< 0.01**	**−0.10 [−0.18 – −0.01]**	**0.02**
Fatty	**4.33 [2.68–5.99]**	**< 0.01**	0.01 [−0.06–0.09]	0.71	0.83 [−0.19–1.85]	0.11	0.76 [−0.25–1.77]	0.14	−0.08 [−0.19–0.02]	0.10	−0.09 [−0.19–0.01]	0.09

*Note:* Bold signifies significant *p*‐value (< 0.05). Visual Analogue Scale questions: ‘Hunger’—how hungry do you feel?, ‘Fullness’—how full do you feel?, ‘Prospective consumption’—how much do you think you can eat?, ‘Thirst’—how thirsty do you feel?, ‘Savoury’—would you like to eat something savoury?, ‘Salty’—would you like to eat something salty?, ‘Sweet’—would you like to eat something sweet?, ‘Fatty’—would you like to eat something fatty?

Abbreviation: 95% CI, 95% confidence intervals.

## 4 Discussion

Our findings reveal that even short‐term behavioural misalignment, akin to night‐shift work, disrupts postprandial glucose and lipid metabolism in ways that mirror early pathophysiological changes in metabolic syndrome. Plasma lipid rhythms shifted in parallel with the 10‐h delay in sleep–wake timing, accompanied by significant amplitude reductions. Night‐shift conditions amplified postprandial glucose and insulin excursions. Importantly, time‐of‐day effects persisted alongside effects of shift condition, strikingly with meals at 18:00 consistently producing the highest glycaemic and insulinaemic responses in both day‐shift and night‐shift conditions.

Statutory UK data indicate that approximately 8.7 million people regularly work evening or night‐time schedules, with 56% of night‐time workers being male and younger adults disproportionately represented (Office for National Statistics 2023). Our participant cohort comprised healthy, young adults (6 men, 3 women). Although generalisability to older or clinical populations is limited, our study design isolates early metabolic responses to circadian and behavioural misalignment relevant to many night workers.

The persistence of elevated 18:00 glucose responses across all shifts confirms previous findings that the circadian rhythm of glucose tolerance is preserved during night‐shifts when behavioural schedules are misaligned (Al‐Naimi et al. 2004; Wehrens et al. 2010; Qian et al. 2018; Centofanti et al. 2024). Qian et al. (2018) observed dynamic β‐cell responsivity to be ~22% lower in the biological evening compared to biological morning, illustrating the independent effect of circadian phase on postprandial metabolism. Postprandial insulin responses did not fully compensate for increased postprandial glucose levels during night‐shifts, supporting reduced insulin sensitivity as the primary driver of postprandial hyperglycaemia (Hampton et al. 1996; Ribeiro et al. 1998; Al‐Naimi et al. 2004; Bescos et al. 2018; Kervezee et al. 2018). This is consistent with findings on the independent effects of circadian misalignment on postprandial metabolism. Morris et al. (2015) reported that circadian misalignment elevated postprandial glucose AUC by ~6% despite a ~14% rise in second‐phase postprandial insulin release, indicating reduced insulin sensitivity as the primary driver of the elevation.

Our data extend these observations by showing that, despite the preserved glucose tolerance rhythm, night‐shift schedules can exacerbate glycaemic responses when multiple, consecutive meals are consumed at inappropriate circadian phases. Our results are consistent with evidence that circadian misalignment reduces insulin receptor activity, downregulates glucose transporters such as GLUT4 and diminishes β‐cell responsiveness during the biological night (Morgan et al. 1998; Takahashi et al. 2018; Speksnijder et al. 2024).

We observed increased insulin rhythm amplitude in NS1 compared to DS and a delayed insulin acrophase during both night‐shifts. McDermott et al. (2024) similarly reported significant delays in glucose and insulin rhythms in constant routine conditions after simulated day‐ versus night‐shifts, alongside altered insulin secretion and disruption of proteins and metabolites involved in insulin regulation, energy metabolism and mitochondrial function.

Night‐shift‐induced misalignment caused substantial phase delays in plasma lipid rhythms, which corresponded to the delayed sleep–wake and mealtimes. These findings reinforce earlier work indicating that peripheral clocks regulating lipid metabolism are more responsive to feeding cues than to central clock signals (which were not shifted in our intervention), leading to circadian decoupling under misaligned conditions (Hampton et al. 1996; Morgan et al. 1998, 2003). The observed amplitude attenuation in lipid rhythms may also contribute to the increased risk of dyslipidaemia and cardiometabolic disease associated with shift work. We observed a further potential effect of circadian decoupling within lipid postprandial dynamics, whereby plasma triglyceride concentrations declined following the last meal since wake (08:00) in NS1.

Several methodological strengths support the current findings. The use of a highly controlled behavioural misalignment intervention with multiple clock‐anchored meals enabled high‐resolution characterisation of metabolic responses across different circadian phases. This design allowed us to disentangle the independent and interactive contributions of endogenous rhythms and behavioural timing, something not possible in simpler two‐meal designs, and supports interpretation of the results within a mechanistic and pathophysiological context.

We showed that altered sleep and meal timing interact with circadian physiology to impair glucose tolerance, blunt lipid rhythmicity and reduce insulin sensitivity. The consistently elevated glycaemic and insulinaemic responses at 18:00 across shift conditions suggest that evening biological timing represents a metabolically vulnerable window. This has potential implications for shift work scheduling, meal timing guidelines and interventions aimed at mitigating cardiometabolic risk in occupational settings (Maury et al. 2010; Maury et al. 2014). Longer‐term studies in real‐world shift‐working populations are needed to determine whether repeated exposure to adverse meal timing leads to sustained metabolic dysregulation. Future work should also examine sex‐ and age‐specific responses, test meal‐timing interventions such as altered distribution of energy intake and assess interactions with sleep duration and light exposure. Linking acute postprandial disturbances to timing and content of individual meals with longer‐term cardiometabolic risk markers will be essential for informing evidence‐based, operationally feasible interventions.

## Author Contributions

Conceptualisation, Debra J. Skene, Cheryl M. Isherwood and Daan R. van der Veen; methodology, Debra J. Skene, Cheryl M. Isherwood and Daan R. van der Veen; investigation, Cheryl M. Isherwood, Ameena M. Khan Sullivan and Hana Hassanin; clinical supervision/oversight, Hana Hassanin; formal analysis, Ameena M. Khan Sullivan, Cheryl M. Isherwood and Daan R. van der Veen; writing – original draft preparation, Ameena M. Khan Sullivan; writing – review and editing, all authors; supervision, Daan R. van der Veen, Cheryl M. Isherwood and Debra J. Skene; funding acquisition, Debra J. Skene, Cheryl M. Isherwood, Daan R. van der Veen.

## Funding

This research was funded by Wellcome (grant number 23704/Z/21/Z). The funders had no role in the design of the study; in the collection, analyses, or interpretation of data; in the writing of the manuscript; or in the decision to publish the results.

## Conflicts of Interest

The authors declare no conflicts of interest.

## Supporting information


**Table S1:** Energy and macronutrient composition of static meal components.
**Table S2:** Energy and macronutrient composition of the flexible meal component for an example portion size (yoghurt).

## Data Availability

The data that support the findings of this study are available from the corresponding author, Ameena M. Khan Sullivan or Daan R. van der Veen, upon reasonable request.
