# Supplementary material for: Diurnal Variation and Impairment of Postprandial Glucose‐Insulin Responses and Lipid Rhythms in Simulated Shift Work
Source: Nutr Bull. 2026 Jun 21;51(3):353–65. doi: 10.1111/nbu.70060 (PMC13522867; doi:10.1111/nbu.70060)
Supplement: Supplementary file 1 [file NBU-51-353-s001.docx]

**Supplementary Material: meal design and protocol**

Throughout the protocol participants ate four identical meals per day based on UK dietary guidelines; 50% carbohydrates (CHO) (15% sugars), 35% fat (11% saturated), and 15% protein (Public Health England, 2016). The meal protocol comprised of readily available palatable vegetarian ingredients with a mixture of textures. Meal descriptions were included in the Participant Information Sheets during recruitment. Individual energy requirements were estimated using the Mifflin St. Jeor equation (Mifflin et al., 1990), adjusted for sex.
Each meal comprised two components. A macronutrient-balanced static component (1499 kcal per day / 4 = 375 kcal), designed to meet the and energy requirements of the participant with the lowest energy requirement and a macronutrient-balanced flexible component, which was used to meet the deficit between the static component and an individual’s total energy requirements.

The static component of each meal comprised of a sandwich (white bread roll, reduced‑fat spread, with chicken‑styled mycoprotein slices, cheese coleslaw with added high‑protein cheese, vegetable oil and pea protein), an orange-flavoured drink, a coconut flavoured biscuit and a malted milk biscuit.

The flexible component was offered as, a yoghurt, consisting of fat‑free Greek yoghurt with added puréed fruits (apples 39%, bananas 32%, strawberries 20%, watermelon juice 5%, raspberries 4%, lemon juice concentrate), icing sugar, double cream, vegetable oil, sucralose, pea protein and maltodextrin.

**Supplementary Table 1: Energy and macronutrient composition of static meal components.**

| Meal component | Portion size (g) | Energy (kJ) | Energy (kcal) | Protein (g) | CHO (g) | Sugars (g) | Fat (g) | Saturated fat (g) | Fibre (g) |
| --- | --- | --- | --- | --- | --- | --- | --- | --- | --- |
| Sandwich | 113 | 1066 | 253 | 12.0 | 28.0 | 2.0 | 10.0 | 3.0 | 3.0 |
| Coconut biscuit | 8 | 168 | 40 | 0.5 | 4.9 | 1.3 | 1.9 | 1.0 | 0.3 |
| Malted milk biscuit | 9 | 184 | 44 | 0.6 | 5.9 | 1.5 | 1.9 | 0.9 | 0.2 |
| Orange juice drink | 200 | 162 | 38 | 0.0 | 8.8 | 8.8 | 0.0 | 0.0 | 0.0 |
| Total per meal | 330 | 1580 | 375 | 13.1 | 47.6 | 13.6 | 13.8 | 4.9 | 3.5 |
| Total per day | 1321 | 6320 | 1500 | 52.4 | 190.4 | 54.4 | 55.2 | 19.6 | 14.0 |

**Supplementary Table 2: Energy and macronutrient composition of the flexible meal component for an example portion size (yoghurt)**

| Meal component | Portion size (g) | Energy (kJ) | Energy (kcal) | Protein (g) | CHO (g) | Sugars (g) | Fat (g) | Saturated fat (g) | Fibre (g) |
| --- | --- | --- | --- | --- | --- | --- | --- | --- | --- |
| Yoghurt | 37 | 257 | 6 | 2.3 | 7.6 | 2.3 | 2.4 | 0.8 | 0.2 |

All meals were consumed in the individual sleep rooms within 14 minutes. Participants were instructed to consume all food provided. If this was not possible (e.g., nausea), the reason for non-compliance was recorded and the leftover food was weighed. This only happened twice out of 153 meals (n = 17 mealtimes during clinical study by 9 participants).

References

Mifflin, M.D., St Jeor, S.T., Hill, L.A., Scott, B.J., Daugherty, S.A. and Koh, Y.O. (1990) “A new predictive equation for resting energy expenditure in healthy individuals,” *The American journal of clinical nutrition*, 51(2), pp. 241–247. Available at: https://doi.org/10.1093/AJCN/51.2.241.

Public Health England (2016) *Government Dietary Recommendations Government recommendations for energy and nutrients for males and females aged 1-18 years and 19+ years*. Available at: https://assets.publishing.service.gov.uk/media/5a749fece5274a44083b82d8/government_dietary_recommendations.pdf (Accessed: August 24, 2023).
